# Transforming Bone Tunnel Evaluation in Anterior Cruciate Ligament Reconstruction: Introducing a Novel Deep Learning System and the TB-Seg Dataset

**DOI:** 10.3390/bioengineering12050527

**Published:** 2025-05-15

**Authors:** Ke Xie, Mingqian Yu, Jeremy Ho-Pak Liu, Qixiang Ma, Limin Zou, Gene Chi-Wai Man, Jiankun Xu, Patrick Shu-Hang Yung, Zheng Li, Michael Tim-Yun Ong

**Affiliations:** 1 Department of Surgery, Faculty of Medicine, The Chinese University of Hong Kong, Hong Kong SAR, China; 2 Department of Orthopaedics and Traumatology, Faculty of Medicine, The Chinese University of Hong Kong, Hong Kong SAR, China

**Keywords:** anterior cruciate ligament reconstruction, tunnel bone analysis system, deep learning, segmentation

## Abstract

Evaluating bone tunnels is crucial for assessing functional recovery after anterior cruciate ligament reconstruction. Conventional methods are imprecise, time-consuming, and labor-intensive. This study introduces a novel deep learning-based system for accurate bone tunnel segmentation and assessment. The system has two primary stages. Firstly, the ResNet50-Unet network is employed to capture the bone tunnel area in each slice. Subsequently, in the bone texture analysis, the open-source software 3D Slicer is leveraged to execute three-dimensional reconstruction based on the segmented outcomes from the previous stage. The ResNet50-Unet network was trained and validated using a newly developed dataset named tunnel bone segmentation (TB-Seg). The outcomes reveal commendable performance metrics, with mean intersection over union (mIoU), mean average precision (mAP), precision, and recall on the validation set reaching 76%, 85%, 88%, and 85%, respectively. To assess the robustness of our innovative bone texture system, we conducted tests on a cohort of 24 patients, successfully extracting bone volume/total volume, trabecular thickness, trabecular separation, trabecular number, and volumetric information. The system excels with substantial significance in facilitating the subsequent analysis of the intricate interplay between bone tunnel characteristics and the postoperative recovery trajectory after anterior cruciate ligament reconstruction. Furthermore, in our five randomly selected cases, clinicians utilizing our system completed the entire analytical workflow in a mere 357–429 s, representing a substantial improvement compared to the conventional duration exceeding one hour.

## 1. Introduction

The anterior cruciate ligament (ACL) is an indispensable soft-tissue structure located within the knee, playing a pivotal role in establishing a connection between the femur and tibia [1]. In cases where the ACL experiences complete rupture, it exhibits a notable deficiency in inherent regenerative capabilities, thus necessitating the requirement for anterior cruciate ligament reconstruction (ACLR) [2]. This surgical intervention is undertaken with the primary objective of restoring and re-establishing stability within the joint, thereby proactively mitigating the potential for damage to both the chondral surfaces and the surrounding tissues.

Ensuring a dependable and standardized evaluation of postoperative knees subsequent to ACLR is of paramount importance, both from a clinical and research standpoint. The cornerstone of ACLR lies in the precise passage of a tendon graft through a bone tunnel, which serves as a replacement for the injured ACL. This tunnel is aptly termed the “graft-bone tunnel”. It has been well-documented that, over time, the femoral and tibial bone tunnels created for graft insertion tend to exhibit an enlargement [3,4,5]. Consequently, numerous studies have embarked on quantitative analyses of these tunnels to gauge post-ACLR recovery, employing metrics such as bone tunnel diameter [6], area [7], and volume [8].

For instance, the work of Peyrache MD et al. delved into an examination of changes in the diameter of the tibial tunnel over time following ACLR using a bone–patellar tendon–bone autograft in a cohort of 44 patients [9]. Their study primarily relied on radiographic assessments to quantify alterations in the geometry of the bone tunnels. However, the landscape of medical imaging has evolved, with computed tomography (CT) emerging as a pivotal player in the evaluation of bone structures and anomalies [10]. CT imaging facilitates the non-invasive assessment of three-dimensional macro- and microstructures in both cortical and trabecular regions, a feat previously attainable only through quantitative histomorphometry of transiliac bone biopsies. The foremost instruments for appraising volumetric density and bone structures include quantitative computed tomography (QCT) and, more recently, high-resolution peripheral quantitative computed tomography (HR-pQCT) [11]. These cutting-edge techniques enable comprehensive evaluations of bone architecture at various skeletal sites within the human body.

In a previous study by Lui PP et al., the investigation aimed to evaluate the relationship between the bone tunnel and ACLR recovery by monitoring changes in bone mineral density (BMD) and the ratio of bone volume to total volume (BV/TV) in a 12-week-old rat model [12]. However, a notable limitation of this study was the utilization of a circular region of interest (ROI) with a diameter of 1.2 mm to demarcate the perimeter of the bone tunnel for assessing the mass and density of the mineralized tissue within the tunnel. However, this approach did not accurately encompass the entirety of the bone tunnel information. Gaining access to the full spectrum of bone tunnel data may provide a significantly more accurate explanation, but the precise and rapid acquisition of comprehensive bone tunnel structure information from medical images emerged as a paramount challenge. The conventional technique employed by the manufacturer, which hinges on the use of a filter and threshold method to define the region of interest [13], was found to be unsuitable for the objective of our study. This is chiefly due to the inherent inaccuracies introduced by the filter and threshold approach in the analysis of bone tunnel information, as it includes undesired bone tissue in the measurements.

In recent years, the field of medical imaging has witnessed a profound transformation owing to the advent of deep learning techniques [14]. Deep learning-based segmentation methods have not only revolutionized the way we process and analyze complex data but also opened up new frontiers in the diagnosis and treatment of various diseases. In pursuit of accurate segmentation of bone tunnels and investigation of their association with surgical recovery post-ACLR, we developed an innovative deep learning-based bone tunnel analysis system. In this system, we employed a Unet-based model for precise segmentation of the bone tunnel within knee CT images. Then, specialized software was applied to achieve a 3D reconstruction of the bone tunnel regions identified by the neural network. Moreover, we also utilized a dedicated extension within this software to conduct a comprehensive analysis of bone texture. Our system can help output some common indicators for evaluating bone in medical reports, such as BV/TV, trabecular thickness (Tb.Th), trabecular separation (Tb.Sp), and trabecular number (Tb.N). Other information, such as the volume of the 3D reconstruction area and the grayscale information of the segmentation result, can also be generated automatically. Our analysis system has demonstrated exceptional stability and speed, showcasing its potential as a valuable tool in the field of medical imaging and orthopedic research.

### 1.1. Clinical Evaluation After ACLfR Surgery

Tunnel widening represents a common phenomenon observed following ACLR. Although the precise causes of tunnel widening remain incompletely elucidated, a range of imaging modalities, including radiographs, CT scans, MRI, and SPECT/CT, have been employed to assess this phenomenon in patients undergoing ACLR.

In an earlier study [15], it was reported that the tunnel size in radiographs can be quantified by measuring the distance between the two sclerotic margins of the tunnel, specifically at 5 mm from the tibial entrance and 5 mm from the tibial exit, perpendicular to the tunnel’s longitudinal axis. Webster KE et al. adopted a different approach, utilizing 1 mm axial CT slices aligned with the axes of the femoral and tibial tunnels to determine the postoperative widths of these tunnels [16]. This assessment was performed on images displaying the widest tunnel dimensions in both sagittal and coronal planes. Tunnel size was expressed in absolute values and relative to the diameter of the drill bit employed during surgery.

In addition to assessing tunnel widening based on tunnel diameter post-ACLR, the work of Frosch KH et al. [17] evaluated changes in bone tunnels by calculating area measurements. Initially, they measured tunnel width perpendicular to the tunnel axis in the sagittal plane. The widest diameter was determined by dividing the tunnel into three equal segments, and the cross-sectional area was subsequently computed. Both absolute and relative values with respect to the drill bit diameter were considered.

However, it is important to note that the methodologies mentioned above may oversimplify the evaluation metrics, potentially resulting in an underestimation of the actual tunnel dimensions. To address this limitation, a more advanced approach using 3D CT images for a more precise assessment of tunnel size was proposed by Araki D et al. [18]. Nevertheless, it is worth mentioning that this method requires manual identification of bone tunnel regions in each image slice, which is a labor-intensive process.

### 1.2. Deep Learning-Based Bone Segmentation

Medical image segmentation has significantly advanced sustainable medical care, which is an emerging biomedical image-processing technology [19]. Deep learning, renowned for its capacity for automatic hierarchical feature representation learning, is increasingly showing its utility in orthopedics, particularly in image segmentation.

For instance, a Unet architecture-based network with innovative data augmentation techniques was designed by Noguchi S et al. [20] to perform bone segmentation on whole-body CT scans. The results of their experiments unveiled a system with outstanding accuracy in detecting and segmenting bony structures, offering potential for use in pathological applications. In [21], the authors sought to create an automated method based on PET/CT scans for quantifying skeletal tumor burden. They introduced a novel neural network architecture for the segmentation of bones in CT scans. Their convolutional neural network (CNN)-based approach was rigorously validated using a separate cohort of 46 patients with prostate cancer, and the same 49 bones were automatically segmented by the CNN in both CT studies from all patients. Their results demonstrated rapid and highly accurate bone volume quantification. In the realm of knee osteoarthritis epidemiology within clinical trials, Liu F et al. developed a fully automated deep learning-based system for the detection of cartilage lesions, offering a high level of diagnostic performance for assessing the knee joint’s articular cartilage [22]. Their innovative deep-learning strategy leveraged a convolutional encoder–decoder network for cartilage and bone segmentation, complemented by a secondary CNN classification network for the detection of structural abnormalities within the segmented cartilage tissue. These pioneering applications of deep learning in medical image segmentation hold significant promise for advancing orthopedic diagnostics and research.

## 2. Materials and Methods

**Figure 1:**
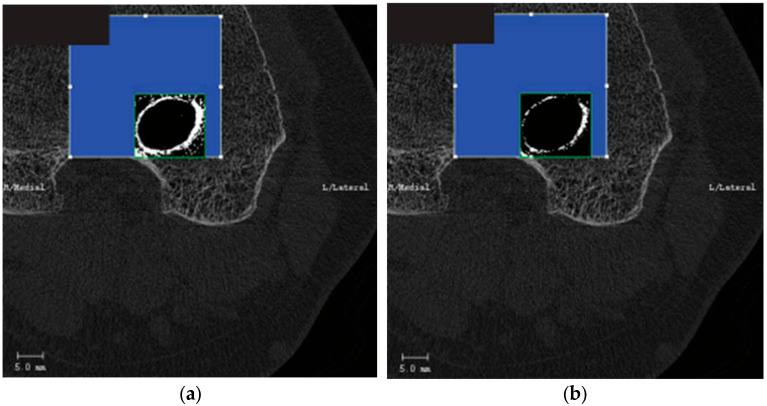
Threshold-based segmentation method. (**a**) Results generated with a low threshold. (**b**) Results generated with a high threshold.

Our research work aims to extract a wealth of information related to bone tunnels within bone grafts to investigate postoperative recovery after ACLR in future studies. Although conventional CT scans routinely provide high-density representations of bony structures, which are distinguishable from soft tissue through thresholding, they tend to lack precision in the segmentation of the bone tunnel, often encompassing unnecessary areas or failing to segment the complete bone tunnel, as shown in Figure 1. Moreover, there are shortcomings in using the software integrated with the imaging device for the selection of the region of interest (RoI). While the surgeon only needs to mark bounding boxes on the initial and concluding images within the selected CT slice sequence, this approach often leads to inaccurate positioning of the automatically generated coordinate frames for the intervening images. As a result, manual adjustments to the RoI become necessary. However, a patient sequence that needs to be tested usually contains hundreds of slices, so it can be timely and expensive for each case.

**Figure 2:**
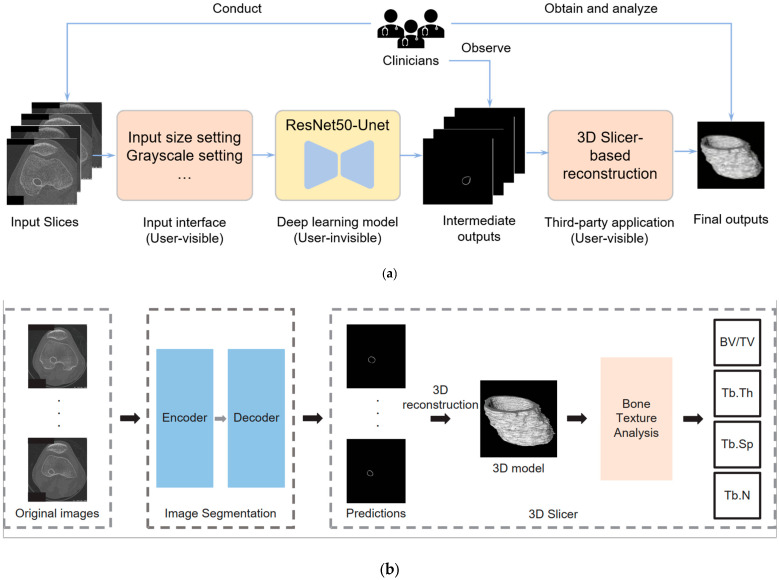
Deep learning-based bone texture analysis system: (**a**) the interaction between clinicians and the system, including data input, observing intermediate outputs, and obtaining and analyzing the final output; (**b**) the technique details of deep learning-based 2D image segmentation and 3D-slicer-based reconstruction.

To surmount these limitations, we present an innovative deep learning-based system designed to automate the segmentation of the bone tunnel. The predictions generated by the neural network serve as the input for subsequent tasks, including 3D reconstruction and comprehensive bone texture analysis. This pioneering methodology elevates the accuracy and efficiency of bone tunnel segmentation within the scope of our study compared with the traditional method. The whole system analysis can be seen in Figure 2. Specifically, Figure 2a demonstrates the interaction between clinicians and the system, encompassing data input procedures, observation of intermediate outputs, and analysis of the final results. Notably, the deep learning module is encapsulated, providing users with input interfaces and output results. Figure 2b presents technical details, where raw slices undergo segmentation by the deep learning model before being reconstructed into 3D structures using the 3D Slicer for subsequent analysis. One advantage of our new bone tunnel texture analysis system is that it can accurately segment the bone tunnel from the original image. Another advantage is that the entire process is very fast. A well-trained operator can complete the analysis of a patient sequence (hundreds of slices) in a few minutes, which would take much longer using traditional methods.

In the initial stage, we leveraged a specialized machine at the Prince of Wales Hospital (PWH), Shatin, Hong Kong, to conduct knee scans on patients who had undergone ACLR surgery, thereby acquiring CT images of these individuals. The specifics of the image acquisition apparatus will be expounded later in this article. Thereafter, we inputted the CT image sequences of the patients, necessitating examination into a meticulously trained Unet-based image segmentation system. The resultant predictive outcomes were subsequently utilized to perform 3D reconstruction within a dedicated software. Building upon the reconstructed data, we further extracted the results of the bone texture analysis, and some indicators for the bone analysis were collected, such as BV/TV, Tb.Th, Tb.Sp, and Tb.N.

### 2.1. XtremeCT

The first-generation HR-pQCT scanner, XtremeCT (Scanco Medical AG, Brüttisellen, Switzerland), boasts the capability to acquire in vivo images at an impressive voxel size of 82 μm (with a resolution < 130 μm). On the other hand, the second-generation HR-pQCT scanner, XtremeCT-II (Scanco), improves the imaging precision further, allowing scans at a voxel size of 60.7 μm (with a resolution > 90 μm) in vivo [23]. This advanced system offers a precise and direct assessment of BV/TV based on segmented images, along with a comprehensive evaluation of trabecular microstructure via distance transform techniques [24,25,26]. In our study, HR-pQCT images were collected by employing the XtremeCT-II scanner at the PWH. When obtaining singular knee images from patients, it is crucial to position the knee accurately within the scanning area. The pertinent scanning parameters, encompassing scan type, layer thickness, and scanning range, are set within the control interface of the XtremeCT-II. As a remark, in order to avoid image blurring or distortion, the patient needs to remain still.

### 2.2. Dataset Information

The availability of an appropriate dataset assumes paramount significance for the image segmentation task, particularly in the context of tunnel bone segmentation post-ACLR. Unfortunately, during the course of the research, no publicly accessible dataset tailored to this specific purpose could be found. To bridge this gap, we first established a new dataset named Tunnel Bone Segmentation (TB-Seg), containing a total of 1058 HR-pQCT scans. These scans, encompassing both femur and tibia components, were judiciously selected from the digital repository housing records of patients who had previously undergone ACLR surgery at PWH, incorporating 493 images of the femur portion and 565 images depicting the tibia segment. We invited expert surgeons to label the regions of interest within each image using an online platform known as Supervisely. To ensure the final training results, we thoughtfully divided it into two distinct sets: one encompassing 793 scans for training purposes and another comprising 265 scans for validation. The adoption of a stratified sampling strategy in this process was instrumental in guaranteeing that each group maintained a balanced number of femur and tibia scans.

### 2.3. Detection and Analysis System

The proposed bone tunnel segmentation system based on deep learning comprises two integral components. Firstly, it leverages a convolutional deep neural network, referred to as the ResNet50-Unet [27], which has been tailored for the segmentation task.

**Figure 3:**
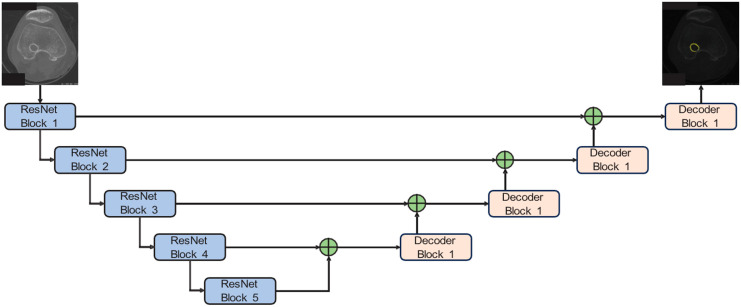
ResNet50-Unet for bone tunnel segmentation. The network integrates a pre-trained ResNet50 encoder with a symmetrical U-net-style decoder. Each arrow represents the direction of data flow through the convolutional blocks.

ResNet50-Unet has a symmetrical structure. It has two components, an encoder and a decoder. These structures are connected to each other by skip connections. In this implementation, we adopt a Unet architecture bolstered by pre-trained ResNet50 weights sourced from PyTorch V.2.5.0, which serves as the backbone for our bone tunnel segmentation model. By integrating residual connections with ResNet into the original Unet framework, it can significantly deepen the encoder portion of the Unet, thereby enhancing its capacity to extract richer information. The network structure of ResNet50 can be seen in Table 1. Each convolution is followed by batch normalization (BN) and the ReLU activation function. These two operations are not represented in Table 1. Meanwhile, the decoder component remains unaltered. Our configuration encompasses five down-sampling and five up-sampling steps, effectively reducing the input image dimensions from 600 × 600 × 3 to a 19 × 19 × 2047 representation and subsequently up-sampling it to yield the original image size. The network structure can be seen in Figure 3.

Regarding the training details, the neural network was trained for 100 epochs, with a batch size set at 12. To optimize the network parameters, we employed the Adam optimizer, initializing it with a learning rate of 0.0001. To further enhance the training efficacy, we incorporated a specialized module for learning rate adjustment, causing it to gradually decrease as the number of iterations advanced. The Adam optimizer is conducted with β_1_ = 0.9, β_2_ = 0.999, and a weight decay of 1 × 10^−10^ during training. Throughout the training and validation phases, we assessed the network’s performance by employing the dice coefficient loss [28], a widely recognized metric in the realm of image segmentation problems. Additionally, after each convolutional layer, we implemented a batch normalization operation [29]. This operation played a pivotal role in normalizing the batch data by adjusting it according to its mean and variance. This normalization process significantly expedited the network’s learning convergence during the training phase. The whole experiment is implemented in PyTorch, deployed on Windows 11 with a GPU of Nvidia GeForce RTX 3060 (12 GB memory).

The second phase of our analysis involves utilizing the prediction results generated by the neural network to conduct an in-depth examination of the bone tunnel texture. To facilitate this, we employed the software tool known as 3D Slicer. Notably, 3D Slicer is an open-source application dedicated to medical image computing. Its development is primarily led by a team of professional engineers working in close collaboration with algorithm developers and domain-specific scientists. As a versatile clinical research tool, 3D Slicer offers a wide array of features, including advanced functionalities like automated segmentation and registration, all of which cater to various application domains. Of particular interest for our study is an extension within 3D Slicer known as the “BoneTextureExtension”, which encompasses several modules designed to enable a comprehensive analysis of bone morphometric characteristics. Within the scope of our research, we harnessed this extension to scrutinize the regions of the tunnel bone that had been predicted by the neural network. Several key indicators, known for their abilities to reflect bone properties, were collected. These indicators encompass BV/TV, Tb.Th, Tb.Sp, and Tb.N.

## 3. Results

### 3.1. The 2D Segmentation

Precision (P), recall (R), intersection over union (IoU), and pixel accuracy (PA) represent the four widely adopted metrics employed to evaluate the training outcomes of the neural network responsible for image segmentation. Precision serves as an effective gauge for the accuracy of positive predictions compared with the ground truth. Recall describes the completeness of positive detections relative to the ground truth. IoU, also recognized as the Jaccard Index, stands as one of the most prevalent metrics in the domain of semantic segmentation. In this analysis, the threshold for accepting accuracy was set at 0.5. Pixel accuracy, on the other hand, quantifies the proportion of correctly classified pixels relative to the total pixel count.

For the results generated by our system, the respective metric values of P, R, mean IoU, and mean PA were 88%, 85%, 76%, and 85%. These values collectively show the overall performance of our dataset trained on ResNet50-Unet, highlighting its ability to accurately predict the bone shell region. However, it is noteworthy that these values, while indicating good training results, are yet to reach the best levels. This observation can be attributed to the following factors.

First, we consider the dataset itself as a potential contributing factor. In this work, it is worth noting that our dataset only contains approximately 1000 scans. Expanding the size of the dataset may yield improvement in training outcomes. Moreover, it is crucial to acknowledge that there are considerable structural variations in the knees of different patients. These variations also extend to the configuration of tunnels formed after ACLR surgery, thus constituting another influential factor affecting the ultimate training results. Additionally, during the dataset labeling process, the delineation of desired areas relies on the expertise of medical professionals. The absence of strict criteria for precisely locating the bone tunnel area somewhat introduces uncertainty to this labeling process, which will also affect the final training and detection results. Furthermore, while the ResNet50-Unet architecture has demonstrated its efficacy in medical image segmentation tasks, employing more intricate neural networks still needs to be considered. Subsequently, we plan to explore the utility of state-of-the-art (SOTA) medical image segmentation models, possibly even developing new models, in the later stages of our research.

**Figure 4:**
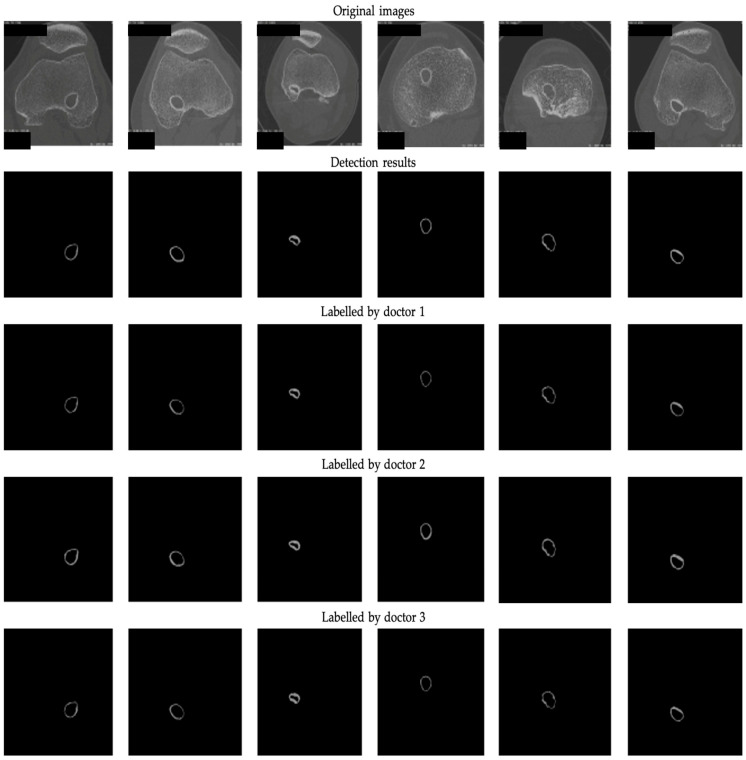
The 2D segmentation results and ground truths labeled by 3 professional doctors.

Figure 4 provides a visual representation of the detection results (DRs) generated by the ResNet50-Unet architecture in conjunction with the results labeled by different expert doctors. Upon a thorough examination of both the figures and the data presented in Table 2, it becomes evident that the ResNet50-Unet architecture exhibits a noteworthy proficiency in accurately segmenting the bone tunnel compared to the results labeled by different doctors. Nonetheless, it is worth noting that the test results of the neural network display notable variations when considering different labeling results.

To further investigate the disparities between the different actual labeling results, i.e., inter-observer variability, we conducted an analysis of the three distinct sets of masks manually labeled by the medical experts, as shown in Table 2. Our analysis revealed that the labeling results of the first and third doctors displayed a relatively high degree of similarity, whereas the second labeling results demonstrated slightly lower congruence with the other two groups. This observation highlights the fact that even when confronted with the same image, individual doctors may make dissimilar choices regarding the regions of interest. The variation in their annotation results can be attributed to differences in experience and, consequently, exert a discernible impact on the outcomes of both the training and the subsequent detection processes. Higher inter-observer variability indicates less consistency among experts regarding the delineation of this structure. Relying solely on annotations from a single expert may constrain the model’s generalizability. In such cases, it is recommended to incorporate consensus from multiple experts through voting or label fusion techniques to establish a more reliable ground truth.

Utilizing the outcomes derived from the neural network’s detection, we also computed the mean grayscale value (mGV) within distinct bone tunnel areas across diverse patients. Given the depiction of tissues as black and bone areas as gray in CT images, the grayscale intensity provides insight into the bone distribution characteristics within the bone tunnel area. Consequently, we documented the grayscale values from all CT slices pertaining to an individual patient, employing the mGV across all slices to encapsulate the whole status of both the tibial and femoral tunnels. Further elaboration on the detailed mGV can be found in Table 3.

### 3.2. The 3D Reconstruction and Bone Texture Analysis

After training the network, we also employed a separate set of 24 patient samples for testing our bone texture analysis system and analyzed the tibial and femoral tunnels of these patients separately. Furthermore, it is worth noting that none of these samples were previously included in the construction of the training dataset. All of the recruited patients had to fulfill the following requirements at the time of testing—undergone ACLR surgery and been within the post-surgery period of 3 to 9 years while actively participating in follow-up appointments at our sports clinic.

In addition, the criteria for subject selection encompasses the following prerequisites: (1) all selected patients must be aged 18 or older; (2) eligible individuals must present with clinically and radiologically confirmed unilateral ACL tear, necessitating primary ACLR. In contrast, the exclusion criteria encompass several factors: (1) patients with concomitant multiple ligament injuries necessitating supplementary surgical procedures; (2) individuals displaying preoperative radiographic signs of arthritis; (3) patients with a history of revision ACL surgery; (4) the presence of femoral tunnel interference screws; (5) contralateral knees afflicted with ACL deficiency or having undergone ACLR; (6) medical co-morbidities; (7) long-term steroid intake; and (8) non-compliance with our rehabilitation protocol.

For a comprehensive examination of bone texture, we employed a specialized software, 3D Slicer, which enabled the transformation of the 2D detection results produced by the Unet model into 3D representations. More specifically, we harnessed a dedicated 3D Slicer extension designed for bone texture analysis. The outcomes of this process are documented in Table 4 for reference and evaluation. These results encompass 3D reconstruction models, as well as essential bone parameters such as BV/TV, Tb.Th, Tb.Sp, Tb.N, and volume information. The images input into 3D Slicer had dimensions of 338 x 338 pixels, and the calculations for BV/TV, Tb.Th, Tb.Sp, and Tb.N were based on this image size. All other parameters were kept at their default values within the software. Regarding volume, the unit of measurement was mm^3^. In summary, our novel system excels in segmenting bone tunnels and producing bone texture indices consistent with those used in clinical medical reports. This achievement holds significant relevance for the subsequent analysis of the relationship between bone tunnel and postoperative recovery following ACLR.

BV/TV expresses the proportion of mineralized tissue inside the reconstructed tunnel. Across 24 patients, femoral BV/TV ranged 0.063–0.199 (mean = 0.134 ± 0.028), whereas tibial BV/TV lay between 0.026 and 0.274 (mean = 0.122 ± 0.048). All values are lower than the 0.20–0.35 interval reported for healthy metaphyseal trabecular bone on HR-pQCT scans [24,25], reflecting post-surgical resorption and remodeling. The highest BV/TV (patient 12 tibia, 0.274) coincided with the greatest trabecular number and the smallest separation, suggesting early bone fill in that tunnel. Femoral trabeculae were marginally thicker (mean = 0.370 ± 0.063 mm) than tibial trabeculae (mean = 0.326 ± 0.061 mm); the overall range (0.199–0.496 mm) aligns with normative data for peripheral cancellous bone [24]. Patients with the highest BV/TV also demonstrated the largest Tb.Th, indicating coupled increases in volume fraction and trabecular hypertrophy.

Tb.Sp showed the widest dispersion of any metric (overall 0.241–1.380 mm). Tibial tunnels displayed greater variability (SD = 0.21 mm) than femoral tunnels (SD = 0.06 mm), consistent with more pronounced and heterogeneous tunnel widening on the tibial side [3]. The outlier value of 1.38 mm (patient tibia 5) coincided with low BV/TV and Tb.N, pointing to extensive trabecular loss. Mean Tb.N was similar for the femur (2.20 ± 0.29 mm^−1^) and tibia (2.22 ± 0.64 mm^−1^), within the normal cancellous range of 1.5–2.5 mm^−1^ reported for the distal tibia [25]. An inverse relationship with Tb.Sp was evident, indicating that loss of trabeculae contributes directly to increased separation. Segmented bone tunnel volume provides a 3D surrogate for overall tunnel size. Femoral tunnels averaged 257 ± 81 mm^3^ (range 53–389 mm^3^); tibial tunnels averaged 195 ± 58 mm^3^ (range 81–290 mm^3^). Despite a wider surgical aperture typically created in the tibia, greater postoperative bone loss may explain the smaller mean tibial volume in this cohort [6].

Table 5 presents the time required to detect patients using our method. Initially, we conducted a brief training session for a doctor unfamiliar with our system to acquaint him with the detection process. Subsequently, we randomly selected samples from five patients for the detection task. The detection process comprises four main stages: image selection, image processing, image segmentation, and 3D Slicer analysis. We counted the time taken for the above process, and the results indicate that the doctor could complete these stages within 10 min. In contrast, traditional methods necessitate the doctor to verify and adjust the selected area for each image, a task that can exceed 1 h. Overall, our system significantly accelerates the bone tunnel analysis process.

Our system still has room for improvement. First, although our dataset addresses the lack of public datasets in this field, its current scale remains suboptimal for robust validation of deep learning-based methods. It would benefit from k-fold cross-validation and external validation in further studies. Second, our two-stage approach—combining deep learning-based segmentation with 3D Slicer-based reconstruction—while offering good interpretability, may lead to loss of inter-slice physical information and increase clinician analysis time. Subsequent efforts should prioritize end-to-end 3D or hybrid 2D/3D fusion models to segment this structure, which could enhance both segmentation accuracy and clinical workflow efficiency.

## 4. Conclusions

To address the limitations of conventional methods in swiftly and accurately acquiring tibial and femoral bone tunnels, we introduce a novel bone texture analysis system rooted in image segmentation. Our system comprises two primary components. First, we feed the image sequence acquired through the HR-pQCT machine into a neural network to detect complete tibial and femoral bone tunnel segments. This phase utilizes the ResNet50-Unet architecture, which has exhibited robust performance in both training and detection. For the subsequent bone texture analysis, we employ an open-source software known as 3D Slicer to accomplish the 3D reconstruction of the segmentation outcomes from the previous step. This process yields the essential indicators used in clinical medical reports for bone analysis. Additionally, we furnish the corresponding reconstructed volume, which augments the information available to doctors for diagnostic purposes.

In order to validate the feasibility of our novel system, we conducted tests on the CT slices of 24 patients who had undergone anterior cruciate ligament reconstruction (ACLR), and we successfully generated the corresponding analysis results. The entire inspection procedure is characterized by its simplicity and speed. We believe that this new system has the potential to become a pivotal tool for healthcare professionals involved in bone tunnel analysis. Furthermore, we introduce a novel TB-Seg dataset specifically designed for bone tunnel segmentation, encompassing over 1000 slices. This dataset represents a pioneering contribution to the field, serving as a valuable resource for further research. In our future work, we aspire to enhance the precision of image segmentation. Moreover, we envision evolving the bone tunnel assessment system into a comprehensive end-to-end solution, correlating clinical outcome data with the imaging pipeline, thereby increasing its accessibility and convenience for surgeons. This design will enable exploratory correlations between early bone tunnel texture signatures and functional recovery, thereby strengthening the translational value of the proposed system and informing personalized rehabilitation protocols.

## Figures and Tables

**Table 1:** ResNet50 neural network architecture (%).

Block	Component	Number
ResNet Block 1	Convolution (kernel size = 7, stride = 2) Max Pooling (kernel size = 3, stride = 2)	x1
ResNet Block 2	Convolution (kernel size = 1, stride = 1)	x3
	Convolution (kernel size = 3, stride = 1)	
	Convolution (kernel size = 1, stride = 1)	
ResNet Block 3	Convolution (kernel size = 1, stride = 1)	x4
	Convolution (kernel size = 3, stride = 2)	
	Convolution (kernel size = 1, stride = 1)	
ResNet Block 4	Convolution (kernel size = 1, stride = 1)	x6
	Convolution (kernel size = 3, stride = 2)	
	Convolution (kernel size = 1, stride = 1)	
ResNet Block 5	Convolution (kernel size = 1, stride = 1)	x3
	Convolution (kernel size = 3, stride = 2)	
	Convolution (kernel size = 1, stride = 1)	

**Table 2:** Comparison of different results (%) regarding (1) detected results (DR) with manual annotations and (2) inter-observer variability. D1: The results labeled by doctor 1. D2: The results labeled by doctor 2. D3: The results labeled by doctor 3.

	Results			Inter-Observer Variability		
Pair	DR–D1	DR–D2	DR–D3	D1–D2	D1–D3	D2–D3
P	90.42	96.45	91.62	98.16	95.21	97.28
R	95.82	89.71	95.16	86.32	93.28	87.25
mIoU	87.63	87.42	88.20	85.31	89.63	85.69
mPA	95.82	89.71	95.16	86.32	93.28	87.25

**Table 3:** Mean grayscale value information.

	1 femur	2 femur	3 femur	4 femur	5 femur	6 femur	7 femur	8 femur
mGV	95.58	77.21	123.65	92.33	118.42	63.70	121.36	104.90
	9 femur	10 femur	11 femur	12 femur	13 femur	14 femur	15 femur	16 femur
mGV	106.93	54.38	114.34	55.24	59.48	118.80	50.76	49.29
	17 femur	18 femur	19 femur	20 femur	21 femur	22 femur	23 femur	24 femur
mGV	97.41	117.54	94.30	110.58	103.88	124.65	73.42	109.97
	1 tibia	2 tibia	3 tibia	4 tibia	5 tibia	6 tibia	7 tibia	8 tibia
mGV	75.22	73.56	105.45	115.49	72.58	58.90	107.86	106.39
	9 tibia	10 tibia	11 tibia	12 tibia	13 tibia	14 tibia	15 tibia	16 tibia
mGV	61.54	87.11	92.82	111.38	70.12	54.64	88.30	90.49
	17 tibia	18 tibia	19 tibia	20 tibia	21 tibia	22 tibia	23 tibia	24 tibia
mGV	104.01	118.83	106.27	102.66	91.23	103.47	97.27	97.07

**Table 4:** The 3D reconstruction and information on bone texture analysis.

	1 femur	1 tibia	2 femur	2 tibia	3 femur	3 tibia
	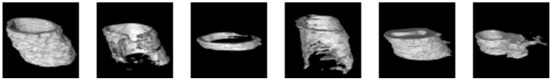					
BV/TV	0.0716	0.0495	0.0623	0.0488	0.1992	0.1721
Tb.Th	0.2668	0.2190	0.2157	0.1985	0.4619	0.3429
Tb.Sp	0.6070	0.7214	0.5647	0.6636	0.3748	0.3226
Tb.N	1.6402	1.3820	1.7641	1.5024	2.6356	3.0671
Volume	243.5359	281.6452	53.3224	287.4996	381.7742	224.1293
	4 femur	4 tibia	5 femur	5 tibia	6 femur	6 tibia
	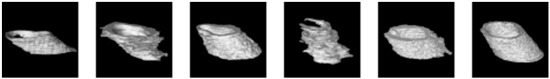					
BV/TV	0.1068	0.1202	0.1349	0.0260	0.1668	0.0912
Tb.Th	0.3310	0.3831	0.3835	0.2197	0.4955	0.3796
Tb.Sp	0.5038	0.5176	0.4613	1.3801	0.4810	0.6769
Tb.N	1.9720	1.9180	2.1500	0.7234	2.0580	1.4692
Volume	218.8271	224.1203	259.8397	81.4737	267.7767	159.2747
	7 femur	7 tibia	8 femur	8 tibia	9 femur	9 tibia
	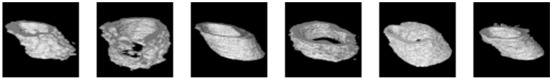					
BV/TV	0.1429	0.1166	0.1309	0.1593	0.1626	0.1293
Tb.Th	0.4680	0.3813	0.3129	0.3706	0.4480	0.3238
Tb.Sp	0.5310	0.5314	0.3879	0.3769	0.4462	0.4066
Tb.N	1.8668	1.8684	2.5571	2.6274	2.2189	2.4399
Volume	303.4012	216.5775	224.0486	261.8792	276.8046	257.3116
	10 femur	10 tibia	11 femur	11 tibia	12 femur	12 tibia
	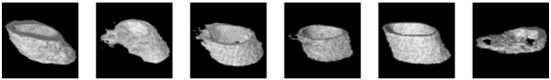					
BV/TV	0.1578	0.1572	0.1350	0.1265	0.1176	0.2744
Tb.Th	0.3918	0.4298	0.3787	0.3521	0.3478	0.4114
Tb.Sp	0.4023	0.4430	0.4552	0.4517	0.4804	0.2412
Tb.N	2.4617	2.2357	2.1789	2.1968	2.0664	4.0759
Volume	302.4606	213.9549	172.3847	159.8268	114.9964	289.7149
	13 femur	13 tibia	14 femur	14 tibia	15 femur	15 tibia
	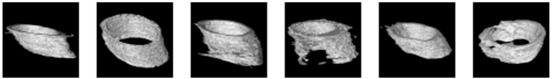					
BV/TV	0.1508	0.0887	0.1412	0.1417	0.1533	0.1183
Tb.Th	0.3358	0.2583	0.3514	0.3566	0.3498	0.2980
Tb.Sp	0.3610	0.4737	0.4038	0.4081	0.3698	0.4093
Tb.N	2.7447	2.0994	2.4552	2.4290	2.6786	2.4255
Volume	315.6059	86.5112	285.6405	207.5080	324.8141	158.0593
	16 femur	16 tibia	17 femur	17 tibia	18 femur	18 tibia
	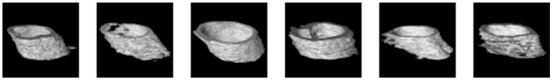					
BV/TV	0.1279	0.1428	0.1209	0.1214	0.1171	0.1204
Tb.Th	0.4100	0.2995	0.3394	0.3400	0.4140	0.3550
Tb.Sp	0.5205	0.3402	0.4558	0.4548	0.5741	0.4788
Tb.N	1.9062	2.9135	2.1778	2.1824	1.7294	2.0730
Volume	389.4614	190.8696	247.7926	120.1776	246.2565	119.0466
	19 femur	19 tibia	20 femur	20 tibia	21 femur	21 tibia
	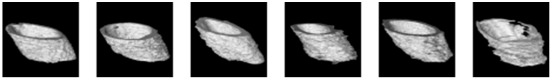					
BV/TV	0.1431	0.1197	0.1351	0.1339	0.1295	0.1378
Tb.Th	0.3955	0.3505	0.3647	0.3490	0.3471	0.2990
Tb.Sp	0.4483	0.4755	0.4379	0.4230	0.4351	0.3517
Tb.N	2.2113	2.0878	2.2648	2.3451	2.2804	2.8193
Volume	273.2562	195.7849	306.7198	220.4959	237.2063	175.8877
	22 femur	22 tibia	23 femur	23 tibia	24 femur	24 tibia
	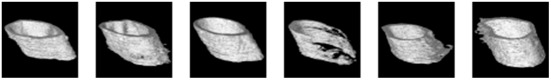					
BV/TV	0.1539	0.1280	0.1113	0.0712	0.1384	0.1256
Tb.Th	0.4124	0.3490	0.3052	0.2326	0.3585	0.3360
Tb.Sp	0.4341	0.4425	0.4454	0.5322	0.4201	0.4342
Tb.N	2.2819	2.2422	2.2298	1.8707	2.3604	2.2854
Volume	376.1578	235.0518	112.3658	173.8293	240.8560	150.8067

**Table 5:** Time for the new system analysis (Sec).

Image Selection	Image Processing	Image Segmentation	3D Slicer Analysis	Total Time
55.87	253.85	81.92	37.84	429.48
50.60	241.58	43.71	45.56	381.45
51.68	256.72	64.24	53.84	426.48
40.60	247.93	63.77	44.95	397.25
30.66	243.83	52.17	31.00	357.66

## Data Availability

The original contributions presented in this study are included in the article. Further inquiries can be directed to the corresponding author.
